# Defining and measuring maternal intentions and practices regarding infant feeding: a scoping review

**DOI:** 10.1186/s12884-026-08768-0

**Published:** 2026-02-11

**Authors:** Yurika Saito, Michiko Nambu, Mari Matsuoka

**Affiliations:** Graduate School of Medicine, Mie University, 2-174 Edobashi, Tsu, Mie Japan

**Keywords:** Infant feeding, Breastfeeding, Mixed feeding, Formula feeding, Maternal intention, Feeding practices, Measurement, Scoping review

## Abstract

**Background:**

Maternal decisions regarding infant feeding are shaped by personal values, social and cultural factors, and healthcare guidance. Although breastfeeding is widely recommended, diverse feeding practices, including mixed and formula feeding, are common across many settings. Previous studies have examined maternal intentions and practices related to infant feeding; however, their conceptual definitions and measurement approaches vary substantially, limiting comparability across studies. The purpose of this scoping review was to systematically map how maternal intentions and practices related to infant feeding have been defined and measured in the existing literature in order to clarify methodological heterogeneity and inform future research design and measurement standardization.

**Methods:**

This review followed a standard methodological framework, adhering to PRISMA-ScR guidelines and the Arksey and O’Malley framework. PubMed, CINAHL, and Ichushi-Web were used for studies published between January 2010 and June 2025. Eligible studies included quantitative, qualitative, and mixed-method studies on maternal intentions or practices regarding breastfeeding, mixed feeding, or formula feeding. Extracted variables included study characteristics, definitions and measurements of intention and practice, timing of assessment, and feeding categories.

**Results:**

Fifty-five studies met the inclusion criteria. Definitions of maternal intention varied widely, ranging from single-item measures for exclusive breastfeeding to validated multi-item scales. Measurement approaches included custom questionnaires, validated scales, qualitative interviews, and single-item assessments, with considerable variations in timing. Practice was most often defined as exclusive breastfeeding; however, operational definitions differed markedly across studies, and only a few studies strictly followed the World Health Organization criteria. Mixed and formula feeding were inconsistently categorized across studies. The intention–practice gap was not specifically examined in any study as a primary outcome. Considerable heterogeneity was observed in measurement timing, feeding classifications, and data sources.

**Conclusions:**

This review highlights substantial variability in the conceptualization and measurement of maternal intentions and practices related to infant feeding. Standardized definitions, consistent feeding classifications, and alignment of measurement timing are necessary for enhancing comparability across studies. Definitive operationalization of the intention–practice gap should be prioritized in future research to advance our understanding of maternal feeding behaviors and inform the development of supportive interventions.

**Supplementary Information:**

The online version contains supplementary material available at 10.1186/s12884-026-08768-0.

## Background

The choice of infant feeding method is shaped by a complex interplay of maternal values, cultural traditions, professional guidance from healthcare providers, and social and environmental factors [1]. The World Health Organization (WHO) and several national health policies have consistently promoted breastfeeding as the optimal method of infant nutrition. However, despite breastfeeding being widely recognized as the biological and public health norm, breastfeeding rates remain suboptimal in many countries, with increasing reliance on mixed feeding and formula feeding observed across diverse settings [2, 3]. Understanding maternal decision-making, particularly the formation of intentions regarding breastfeeding and the translation of those intentions into practice, is essential for contextualizing the increasing diversity of infant feeding choices. Previous studies have captured the constructs of “intention” and “practice” in infant feeding from multiple perspectives. However, the definitions and measurement approaches used to operationalize these concepts vary substantially across studies, hindering conceptual consistency and cross-study comparisons [4, 5]. While internationally recognized definitions for infant feeding practices are available, particularly through WHO guidance, their application and measurement remain heterogeneous across studies. In contrast, although several validated instruments to assess maternal feeding intentions have been developed, no universally accepted definition or standardized measurement approach exists, resulting in considerable heterogeneity in how intentions are conceptualized and assessed. For example, “intention” has been assessed either as a single-item measure reflecting mothers’ prenatal feeding preferences [6–8] or as a composite attitudinal score derived from validated multi-item scales [9–11]. Similarly, “practice” has been evaluated using diverse indicators, such as breastfeeding initiation and duration, and the proportion of breast milk to formula feeding [12, 13]. Notably, most previous reviews were primarily focused on breastfeeding; only a few comprehensively addressed mixed or formula feeding in the context of infant nutrition [14, 15].

Clarifying these variations across studies is essential for establishing a foundation for future research design and evaluation of breastfeeding support programs. To achieve this, a comprehensive understanding of how “intention” and “practice” have been defined and measured in previous studies, regardless of the methodology applied (quantitative or qualitative), is warranted. Therefore, the purpose of this scoping review was to systematically identify and map the conceptualization and measurement of maternal “intentions” and “practices” regarding infant feeding. By synthesizing the findings from previous studies, we sought to elucidate conceptual frameworks and methodological trends in this field, thereby providing a basis for future empirical research and the development of supportive interventions.

## Methods

This scoping review was conducted to comprehensively explore and map the definitions and measurements of maternal intentions and practices related to infant feeding as reported in the existing literature. The review was conducted in accordance with the standardized methodological framework outlined in the PRISMA-ScR guidelines [16] and the framework proposed by Arksey and O’Malley [17]. The review protocol was prospectively registered prior to data extraction (University Hospital Medical Information Network, UMIN000058048).

### Identifying the research question

This review was guided by the following primary research questions:

1. How have maternal intention and practice been defined and measured in previous studies?
2. How have breastfeeding, mixed feeding, and formula feeding been defined, and in what contexts and outcomes have they been examined?

The PubMed, CINAHL, and Ichushi-Web databases were searched to identify relevant studies. The search covered articles published between January 1, 2010, and June 2, 2025. We limited the search to studies published from 2010 onward because definitions of infant feeding categories (e.g., exclusive breastfeeding) and related research methods were standardized and widely disseminated following the global uptake of WHO recommendations in the late 2000s. The full electronic search strategy for each database is provided in Appendix 1.

### Eligibility criteria

Studies were included if they met all of the following criteria:

- Participants were pregnant or postpartum mothers
- The study involved the analysis of maternal intentions or practices regarding infant feeding methods
- Cohort studies, cross-sectional studies, interventional studies, qualitative studies, or case series
- Articles published in English or Japanese

Studies that exclusively evaluated the effectiveness of breastfeeding support programs, as well as those limited to the evaluation of twin pregnancies or mothers with specific medical conditions or backgrounds, were excluded. These studies were omitted because their findings were dependent on specific interventions or contextual factors, making them unsuitable for capturing the conceptualization and measurement of “intention” and “practice.”

### Study selection

The study selection process was conducted in two stages. The titles and abstracts of the articles were screened in the first stage, and a full-text review was conducted in the second stage. Study selection in both stages was conducted independently by two reviewers (YS and MN). Discrepancies between them were resolved through discussion, and when consensus could not be reached, a third reviewer was consulted.

### Charting the data

A standardized data extraction form was developed to collate data on key study characteristics, including name of the first author, year of publication, country of study, language, journal, study design, research aim, participant details (sample size and study period), definitions and measurement approaches used for the evaluation of intention, methods used for the assessment of practice, related outcomes, types and definitions of feeding methods, and main findings. After data extraction, the collated information was reviewed and verified by an independent researcher (YS and MN). The data extraction framework used in this scoping review is provided in Appendix 2.

### Collating, summarizing, and reporting the results

The included studies were first categorized according to infant feeding practices (e.g., exclusive breastfeeding, mixed feeding, or other feeding categories) that were explicitly defined and measured rather than by study outcomes. Subsequently, the studies were further organized according to study design and summarized in a tabular form. Finally, data on the definitions and measurement approaches used for the evaluation of intention, practice, and the intention–practice gap in each study, along with related outcome indicators, were extracted and systematically charted in tables. In line with the methodological framework for scoping reviews, no assessment of study quality or risk of bias was performed.

### Patient and public involvement statement

Patients and members of the public were not involved in the design of this study, the data collection and analysis, preparation of the manuscript, or the decision to publish.

## Results

### Study characteristics

**Fig. 1:**
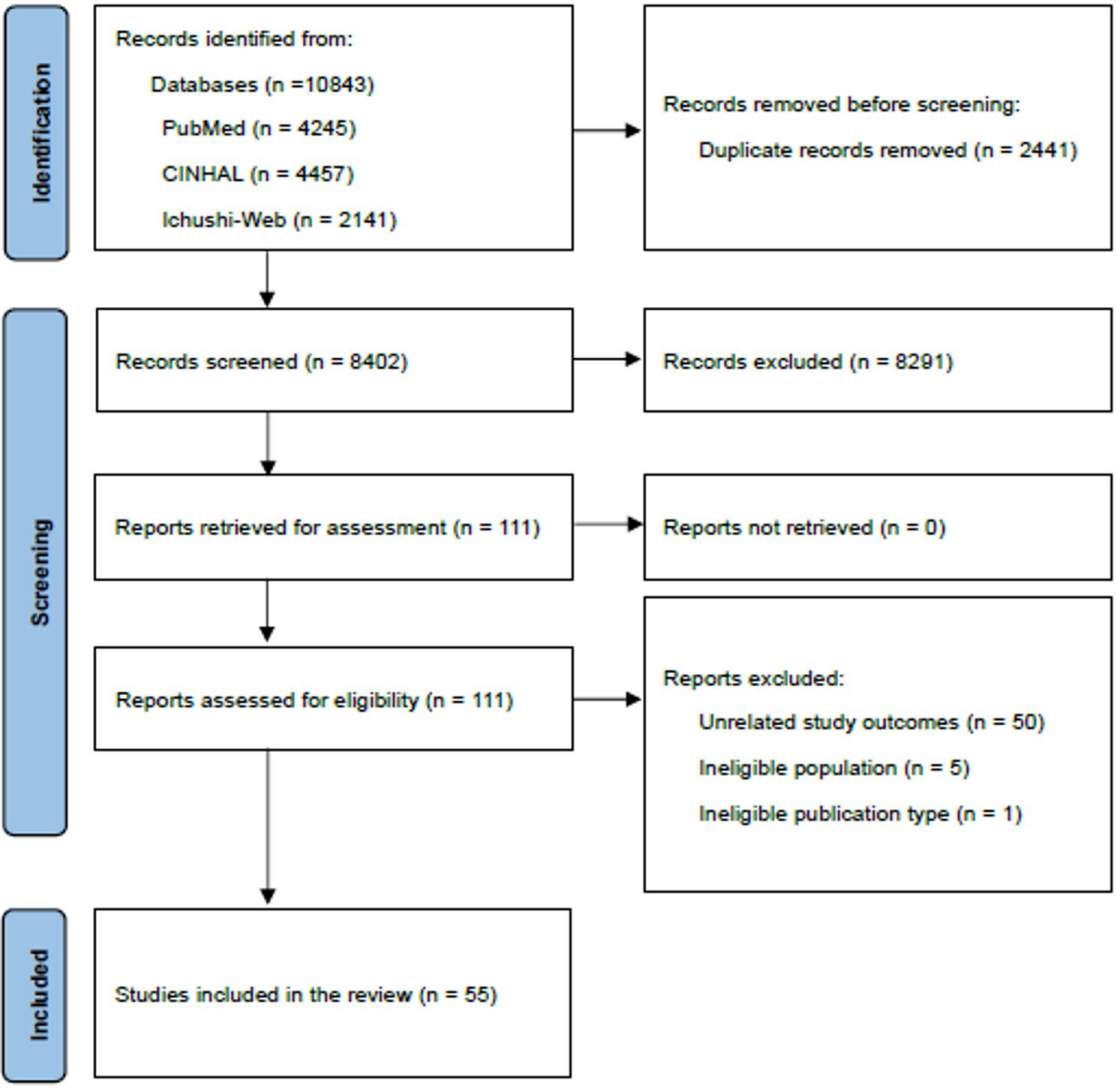
PRISMA flow diagram

The screening process employed in this review is illustrated in Fig. 1. A total of 10,843 records were initially identified based on the eligibility criteria. After removing 2,441 duplicates, 8,402 titles and abstracts were screened, and 8,291 records were excluded. The full texts of the remaining 111 articles were then assessed for eligibility. Of these, 56 were excluded for the following reasons: unrelated study outcomes (*n* = 50), ineligible population (*n* = 5), and ineligible publication type (*n* = 1). Consequently, 55 studies met the inclusion criteria and were included in this review.

The characteristics of the studies included in this review are summarized in Table 1. Of the 55 studies, 15 (27%) were published between 2010 and 2015 [3, 6, 9, 13, 18–28], 20 (40%) between 2016 and 2020 [4, 5, 7, 10, 11, 29–43], and 18 (33%) between 2021 and 2025. Regarding design, 23 studies (42%) were cross-sectional [6, 7, 9–11, 20, 27, 30, 31, 34–36, 39, 41, 43–51], 7 (13%) were non-randomized controlled trials [32, 33, 36, 37, 45, 52, 53], 5 (9%) were mixed-methods studies [4, 6, 18, 52, 54], 5 (9%) were qualitative studies [3, 26, 40, 55, 56], 4 (7%) were randomized controlled trials [24, 40, 42, 57], 1 (2%) was a case–control study [43], and 1 (2%) was an observational study [58]. Regarding the study locations, 25 studies (46%) were conducted in the United States [9, 10, 25, 27, 29, 32, 33, 35, 36, 38, 42, 43, 45–49, 51–53, 55, 57–60], 9 (16%) in the United Kingdom [7, 10, 18, 23, 25, 26, 31, 48, 51], 5 (9%) in Australia [30, 47, 53, 54, 61], 2 (4%) in Canada [27, 45], and 2 (4%) in Jordan [11, 50]. One study was conducted in each of the remaining countries (2%). The median sample size of the studies was 200 (interquartile range [IQR] = 52–445; range = 1–21,842). Regarding the timing of participant inclusion, 9 studies (16%) were focused on the antenatal period only [9, 20, 23, 25, 46, 47, 51, 57, 59], 13 (24%) were focused on the postnatal period only [6, 7, 11, 26, 27, 31, 34, 36, 37, 40, 48, 53, 56], and 22 (40%) involved analyses of longitudinal data obtained during the antenatal and postnatal periods [7, 18, 19, 21, 22, 28, 29, 32, 33, 35, 37–39, 41, 43, 47, 49, 51, 52, 55, 60, 61]. The timing of participant inclusion was not specified in 11 studies (20%). In studies that included detailed information on postpartum timing, the median postpartum duration reported was 6.0 months (IQR = 3.0–6.0).

**Table 1:** Characteristics of the included studies

Study characteristics	*n* (%)
Year of publication	
2010–2015	15 (27)
2016–2020	20 (40)
2021–2025	18 (33)
Study design	
Cross-sectional study	23 (42)
Nonrandomized controlled trials	7 (13)
Mixed-methods study	5 (9)
Qualitative study	5 (9)
Randomized controlled trials	4 (7)
Case-control study	1 (2)
Observational study	1 (2)
Country	
United States	25 (46)
United Kingdom	9 (16)
Australia	5 (9)
Canada	2 (4)
Jordan	2 (4)
American Samoa	1 (2)
Bangladesh	1 (2)
Hong Kong China	1 (2)
Iran	1 (2)
Jamaica	1 (2)
Japan	1 (2)
Kenya	1 (2)
Malaysia	1 (2)
Oman	1 (2)
Rwanda	1 (2)
Saudi Arabia	1 (2)
Spain	1 (2)

The definitions, measurement methods, and timing of assessment for maternal intentions regarding infant feeding are summarized in Table 2. The most common definition of intention was “intending to exclusively breastfeed,” which was reported in 26 studies (47%) [4, 6, 7, 9, 10, 19–22, 24, 26, 29, 31, 34, 36–39, 41, 44, 48, 49, 51, 54, 58, 62], followed by definitions encompassing “any breastfeeding,” reported in 19 studies (35%) [10, 21–23, 25–27, 30, 36, 38, 39, 43, 49–51, 55, 56, 58], and “intending to mix or formula feed,” reported in 8 studies (15%) [6, 22, 23, 29–31, 36, 59]. Intentions related to the duration of breastfeeding (i.e., planned length of feeding) were reported in 13 studies (24%) [5, 9, 27, 28, 35, 38, 47, 49, 60]. Regarding measurement methods, custom-designed questionnaires were used most frequently (19 studies, 35%) [4, 6, 7, 19–21, 29–37, 44, 55, 57, 59], followed by validated scales (12 studies, 22%) [9–11, 18, 20, 25, 28, 41, 50, 51, 54, 62, 63]. Among the validated scales, the Infant Feeding Intention Scale was used in seven studies (58%) [9, 10, 41, 51, 54, 62, 63], Theory of Planned Behavior (TPB)–based instruments in three studies (20%) [4, 18, 28], and intention-related items from the Iowa Infant Feeding Attitude Scale in two studies (17%) [25, 50]. Qualitative interviews or focus groups were used in nine studies (16%) [3, 4, 6, 26, 37, 40, 48, 55, 56], and other or unspecified approaches were employed in five studies (9%) [22, 29, 33, 59, 61]. Four studies (7%) [27, 38, 50, 58] involved the assessment of intention using a single-item question. Given that multiple definitions or measurement approaches were applied in several studies, the total number of classifications exceeded the number of included studies.

**Table 2:** Summary of definitions and measurement methods used for evaluating maternal intentions regarding infant feeding

Definition of practice	Duration-based	Medical records	Self-report / Questionnaire	Other / Unclear	Total
Exclusive breastfeeding	5 (24%)	3 (14%)	2 (10%)	11 (52%)	21 (38%)
Mixed feeding	1 (7%)	2 (14%)	2 (14%)	9 (64%)	14 (26%)
Any/Predominant breastfeeding	0 (0%)	1 (20%)	1 (20%)	3 (60%)	5 (9%)
Other/Unspecified	2 (13%)	0 (0%)	6 (40%)	7 (47%)	15 (27%)
Total	8 (15%)	6 (11%)	11 (20%)	30 (55%)	55 (100%)

The definitions and measurement approaches used in the evaluation of practices related to infant feeding are summarized in Table 3. Representative studies are presented in the table, whereas the complete list of all 55 studies is provided in Additional file 1.

**Table 3:** Definitions and measurements of infant feeding practices in the included studies

Author (Year)	Definition of practice	Reference/Criteria used	Measurement method	Measurement timing	Outcome indicator(s)
Ahishakiye et al. [4]	Exclusive breastfeeding	WHO-based definition	Maternal self-report interviews	1, 3, 6 months postpartum	Continuity of EBF
Nelson et al. [5]	Exclusive/any breastfeeding	Not specified	Self-administered questionnaire	Prenatal to 6 months postpartum	Intended vs. actual duration
Fallon et al. [31]	Exclusive vs. mixed feeding	Self-defined	Online survey + qualitative interview	0–6 months postpartum	Feeding mode (exclusive/ combination/ formula)
Bernard et al.[29]	Exclusive breastfeeding	WHO-based	Self-report questionnaire	3 months postpartum	EBF rate
Al-Sagarat et al. [11]	Any breastfeeding	Self-defined	Structured interview	6 weeks postpartum	Breastfeeding initiation and duration
Holton et al. [54]	Exclusive/ partial breastfeeding	WHO-based	Questionnaire + interview	Prenatal and 6 months postpartum	Continuity of breastfeeding by BMI group
Anderson et al. [49]	Exclusive breastfeeding	Not specified	Structured maternal interview	1, 3, 6 months postpartum	Duration of EBF
Papadopoulos et al. [14]	Mixed milk feeding	Newly defined “mixed feeding index”	24-hour recall diary	0–12 months postpartum	Feeding pattern score
Aranda et al. [57]	Exclusive/any breastfeeding	Self-defined	Medical record + self-report	Discharge and 3 months postpartum	EBF during hospital stay
Onifade et al. [61]	Any/EBF	WHO-based	Medical records	Discharge, 3, 6 months postpartum	EBF rate by model of care
Bahorski et al. [60]	Intended vs. actual feeding plan	Self-defined	Questionnaire	Birth to 3 months postpartum	Deviation from the intended plan

*EBF* exclusive breastfeeding, WHO-based, aligned with the WHO definition; self-defined: author-defined criteria

The most common definition of practice was “exclusive breastfeeding,” which was used in 21 studies (38%) [4, 6, 7, 9, 18–22, 24, 30, 31, 33, 34, 36, 37, 44, 54, 57, 59, 60]. Multiple classifications that included “mixed feeding” [3, 6, 7, 18, 31, 33, 36, 39, 40, 44, 48, 52, 56, 60] or “any/predominant breastfeeding” were employed in several studies [7, 20, 21, 30, 57]. The operational definition of exclusive breastfeeding varied considerably across studies; only a few specifically referred to the WHO definition.

For this review, classifications such as exclusive breastfeeding, mixed feeding, and any/predominant breastfeeding were determined based on the descriptions of infant feeding practices reported in each study. Studies that involved evaluation of combinations of breastfeeding and formula feeding or quantification of proportions of breast milk were categorized according to the definition and contextual information most closely aligned with their reported data.

Various measurement methods were identified in the reviewed studies. These included self-reported questionnaires or surveys [5, 11, 26–29, 35, 40, 45, 55, 60], extraction from medical records [9, 24, 33, 52, 59, 61], and assessments of breastfeeding duration (e.g., 6 or 12 months) [4, 6, 21, 32, 48, 49, 54]. However, detailed descriptions of definitions or measurement approaches were not provided in several studies [7, 30, 31, 44, 57, 62] (Table 3).

Among the included studies, definitions of infant feeding practices varied substantially.

Exclusive breastfeeding defined according to the WHO criteria was reported in 15 studies (27%), mixed feeding was explicitly defined in 12 studies (22%), and formula-only feeding was defined as a distinct category in 8 studies (15%).

Regarding data sources used to assess infant feeding practices, self-reported questionnaires or surveys were used in approximately 60% of studies, medical record data in 25%, and a combination of data sources in 15%.

Among studies that reported the timing of postpartum assessment, the median timing of measurement was 6 months postpartum.

## Discussion

This scoping review revealed substantial heterogeneity in how maternal intentions and practices regarding infant feeding have been defined and measured across studies. In particular, the concept of “intention” ranged from a single-item question assessing whether the mother planned to exclusively breastfeed [29, 30] to the use of multi-item validated instruments such as the Infant Feeding Intention Scale [54, 62] and questionnaires informed by TPB constructs [4]. This diversity in maternal intentions toward infant feeding reflects the influence of cultural, social, and individual value systems on maternal decision-making regarding breastfeeding, as reported in previous studies [20, 30]. However, the lack of consistency in indicators and timing of measurements complicates cross-study comparisons and meta-analyses. Therefore, future research should prioritize clearer conceptualization and validation of measurement tools for maternal feeding intentions. In contrast, research on infant feeding practices would benefit from further emphasis on the consistent application and transparent reporting of existing standard definitions, such as those proposed by the WHO.

Regarding infant feeding practices, the term “exclusive breastfeeding” was used in most studies; however, its specific definition varied widely across studies. The WHO criteria for exclusive breastfeeding were adopted in a few studies [7], whereas self-defined classifications were used in many studies [21, 22, 36]. In some studies, breastfeeding was further categorized based on breastfeeding duration or the proportion of breast milk fed to the infant [23, 32]. Data collection methods also varied across studies. The methods employed included the use of self-reported surveys [55], extraction from medical records [9, 61], and assessments based on breastfeeding duration [38]. Such methodological variation enables adaptation to different research aims and populations but limits comparability and policy applicability. Therefore, transparency in data collection procedures should be ensured in future research, and the definitions and methodologies used should be aligned with the definition of “practice” proposed in the WHO recommendations.

Most previous studies were designed around breastfeeding, with relatively few treating mixed feeding or formula feeding as independent categories [10, 36]. However, a considerable proportion of mothers practice a combination of breastfeeding and formula feeding in clinical and community contexts [30]. This highlights the need for an inclusive assessment approach that captures maternal flexibility and support needs [48]. Thus, future research should be designed to comprehensively capture the full spectrum of infant-feeding practices rather than focus solely on the promotion of breastfeeding.

Consistent with the findings of previous reviews, the present review showed that self-report questionnaires were the most frequent data-collection method used in the analyzed studies. Although the use of self-report questionnaires is cost-efficient, it is prone to social desirability bias [64] and recall bias [65]. In contrast, studies conducted using medical record data offer greater objectivity; however, they are limited by issues related to recording accuracy and inconsistent definitions [66]. Integrating multiple data sources and using longitudinal designs to track temporal changes may help overcome these challenges. In addition, mixed-methods approaches should be adopted in future studies to depict the transition from intention to practice more precisely. Furthermore, further research is necessary for the development of theory-driven instruments grounded in frameworks such as the TPB or the Self-Efficacy Model.

This review represents the first comprehensive attempt to systematically map the definitions and measurement methods used for the evaluation of maternal intentions and practices regarding infant feeding. The findings of this review, obtained by synthesizing evidence from various studies published since 2010, highlight the methodological heterogeneity across studies and underscore the need for conceptual and procedural integration. Specifically, the results of this review indicate three priorities in future research: (1) standardization of the definitions of intention and practice; (2) incorporation of inclusive perspectives that encompass mixed and formula feeding; and (3) validation of measurement reliability and methodological rigor.

In reviewing how maternal feeding intentions and practices have been defined and measured, we noted that although many studies assessed intentions and practices within the same research framework, no study explicitly defined or operationalized the relationship between intentions and practices as a primary outcome. Given that the intention–practice gap is a key behavioral mechanism through which early expectations translate, or fail to translate, into real-world feeding outcomes, establishing a clear and standardized definition is essential for generating comparable evidence and identifying precise intervention targets. Some studies highlighted certain discrepancies, such as whether mothers achieved their intended duration of breastfeeding [23] or whether the planned and actual feeding modes differed [52]; however, their indicators lacked systematic structure. In addition, variability in the following factors were observed: (a) operationalization of the intention–practice gap (e.g., binary classification of goal achievement vs. non-achievement, quantitative deviation scores, or ratio-based comparisons); (b) alignment of measurement timing (prenatal intention vs. postnatal practice); (c) classification schemes that incorporated mixed feeding; and (d) integration of psychosocial and structural factors such as self-efficacy, workplace support, socioeconomic status, smoking, and partner involvement. These variations indicate that the following key factors should be considered in future research: (1) standardized operational definitions of the intention–practice gap (e.g., continuous deviation from the intended feeding mode or binary “intention achieved” indicators); (2) prospective temporal alignment of prenatal and postnatal data; (3) multilevel classification that includes mixed feeding; (4) analytic linkage of psychosocial and structural determinants; and (5) longitudinal and triangulated study designs that allow for identification of modifiable determinants and intervention targets with greater precision.

The major strength of this scoping review is that it involved systematic inclusion of diverse studies published after 2010 and comprehensive mapping of definitions and measurement approaches used for the analyses of both intention and practice. Unlike previous reviews that primarily emphasized the promotion or outcomes of breastfeeding, the present review was focused on the conceptual and methodological foundations of infant-feeding research.

Nevertheless, this review has some limitations that should be noted. First, the literature search was limited to English- and Japanese-language publications. Inclusion of reports published in other languages may reveal alternative conceptualizations. Second, consistent with the nature of scoping reviews, no assessment of methodological quality or risk of bias was performed in this review. Third, heterogeneity and inconsistency in the definitions of “intention,” “practice,” and “mixed feeding” across studies may have introduced some subjectivity during data categorization. In addition, substantial heterogeneity in the definitions and measurement of infant feeding intentions and practices limited not only cross-study comparability but also the feasibility of evidence synthesis, such as inclusion in meta-analyses, thereby constraining the generation of comprehensive and cumulative evidence in this field.

Despite these limitations, this review provides a comprehensive overview of existing evidence and establishes a foundation for standardization and conceptual advancement in future research on maternal infant-feeding intentions and practices.

## Conclusions

This scoping review systematically mapped how maternal intentions and infant feeding practices have been defined and measured in studies published since 2010. The findings revealed substantial methodological heterogeneity in the measurement of maternal intentions and in the operationalization of infant feeding practices, particularly exclusive breastfeeding, with inconsistent definitions and limited application of the WHO criteria. In addition, mixed feeding and formula feeding were infrequently treated as distinct categories.

Future research should prioritize the development of standardized and transparent definitions, adopt longitudinal designs that capture changes in intentions and practices over time, and apply WHO-consistent, multi-level classifications that reflect the full spectrum of infant feeding behaviors. Such efforts will strengthen the comparability of studies and advance evidence-based research on maternal and infant feeding. 

## Supplementary Information

Supplementary Material 1. Summary of the definitions and measurements of infant feeding practice employed in the included studies. This table provides a complete list of the definitions and measurement approaches used for the evaluation of practices related to infant feeding in all 55 reviewed studies.

Supplementary Material 2.

Supplementary Material 3.

## Data Availability

All data extracted and analyzed during the review are included in this published article (and its supplementary information files). Additional information is available from the corresponding author upon reasonable request.

## Abbreviations

TPB: Theory of Planned Behavior
WHO: World Health Organization
