# Supplementary material for: Defining and measuring maternal intentions and practices regarding infant feeding: a scoping review
Source: BMC Pregnancy Childbirth. 2026 Feb 11;26:286. doi: 10.1186/s12884-026-08768-0 (PMC12998363; doi:10.1186/s12884-026-08768-0)
Supplement: Supplementary file 1 — Supplementary Material 1. Summary of the definitions and measurements of infant feeding practice employed in the included studies. This table provides a complete list of the definitions and measurement approaches used for the evaluation of practices related to infant feeding in all 55 reviewed studies. [file 12884_2026_8768_MOESM1_ESM.docx]

**Additional file 1.** Summary of the definitions and measurements of infant feeding practices employed in the included studies

| Author (Year) | Type of feeding | Definition of practice | Outcome indicator(s) | Main findings |
| --- | --- | --- | --- | --- |
| Holton et al. (2024) | Exclusive breastfeeding only | Exclusive breastfeeding for the first 6 months | Exclusive breastfeeding rates at discharge and 6–8 weeks postpartum (e.g., 61.0%, 53.7%) | Women with higher BMI reported barriers such as latch issues, pain, and returning to work. No significant differences in intention or initiation according to BMI. Midwives noted unrealistic expectations among high-BMI women. |
| Barwani et al. (2023) | Measured based on “intensity” (0–100% breastmilk) | Breastfeeding intensity = (breastmilk feedings) / (total feedings in 24h) | Breastfeeding intensity (percentage of feedings with mother’s milk in a 24-hour recall at 8 weeks postpartum) | Feeding intention predicted breastfeeding intensity. Subjective norms (social/professional support) were strongest predictors of intention. Return to work/school negatively impacted intensity |
| Bernard et al. (2020) | Exclusive breastfeeding, partial breastfeeding, exclusive formula feeding | Based on self-report; categories merged into three levels: exclusive breastfeeding, partial breastfeeding, exclusive formula feeding | Actual infant feeding practice at birth and 3 months postpartum | Catholic women were significantly more likely to intend to practice and actually practice exclusive formula feeding than unaffiliated mothers. Differences remained after adjustment |
| Sartor et al. (2024) | Exclusive breastfeeding, mixed feeding, exclusive formula feeding | Based on actual feeding types recorded in EMR during hospital stay | Exclusive breastfeeding during hospital stay, proportion of breastfeeding, time to first feed, time to first breastfeed | Intention to breastfeed and race were the strongest predictors of exclusive breastfeeding. Black women had significantly lower intention and practice rates. Early skin-to-skin contact and timing of first feeding were associated with higher breastfeeding rates |
| Kuswara et al. (2020) | Exclusive breastfeeding, mixed feeding (breast + formula), formula only, introduction of other fluids and solids | WHO definition of EBF (only breast milk, expressed milk, vitamin drops/medicine; no other fluids/foods) | Exclusive breastfeeding (EBF) at 4 months, introduction of formula by 1 month, any breastfeeding up to 12 months | 93% initiated breastfeeding. EBF declined to 44% at 1 month, 33% at 4 months, and 18% at 6 months. Formula use was 55% at 1 month and 63% at 6 months. Intention to mix feed, low awareness of guidelines, and low self-efficacy were significant predictors of lower EBF rates. |
| Linares et al. (2014) | Exclusive breastfeeding, breastfeeding with formula (BF-F), formula only | EBF: breastmilk only during hospital stay  BF-F: breastfeeding with some formula  Data from hospital lactation records | Exclusive breastfeeding status at hospital discharge | Higher IFI score, vaginal delivery, and higher pregnancy acceptance rate predicted EBF at discharge. IFI score was positively associated with EBF |
| Fallon et al. (2017) | Exclusive formula feeding, combination feeding (various degrees), exclusive breastfeeding (prenatal intention only, not in current sample) | WHO-defined categories of infant feeding (exclusive breastfeeding, combination feeding, exclusive formula feeding) | Current feeding type: exclusive formula feeding from birth (EFF), from exclusive breastfeeding to exclusive formula feeding (EBF→EFF), or combination feeding (combi) | 67% reported guilt, 68% reported stigma, 76% reported the need to defend their choice. Mothers with the intention to exclusively breastfeed had to practice formula feeding (EBF→EFF) experienced the highest guilt and dissatisfaction. Mothers who intended to practice exclusive formula feeding reported higher stigma levels. Internet (31%) was most common source of information; Only 23% sought help from health professionals. Support from professionals was generally low-to-moderate. |
| Hawley et al. (2015) | Exclusive breastfeeding, mixed feeding (breast + formula), formula only (backup option) | WHO recommendation referenced (exclusive breastfeeding for 6 months). Formula described as supplement or “backup option” | Reported feeding practices at 3 and 8 weeks postpartum: exclusive breastfeeding, mixed feeding, or formula supplementation | 11/12 mothers intended to practice EBF, but 9/12 were supplementing with formula by 3 weeks postpartum. Barriers included perception of insufficient milk, breastfeeding pain, convenience of formula feeding, and reliance on family advice. Benefits of breastfeeding well recognized but exclusivity undervalued. |
| Ahishakiye et al. (2020) | Exclusive breastfeeding, breastfeeding with herbal/traditional remedies, formula/mixed feeding | Exclusive breastfeeding defined as feeding only breastmilk with no other food/liquid for 6 months | Exclusive breastfeeding at 1 week, 4 months, and 6 months postpartum | Though 82% of the mothers intended to practice EBF for 6 months, only 31% practiced it. Barriers included perceived insufficiency, infant cues, social norms, poverty, workload. Facilitators included knowledge, confidence, and health worker and family support |
| Komninou et al. (2017) | Exclusive breastfeeding, combination feeding (various degrees of formula supplementation) | WHO-defined categories, stratified into two groups for analysis: EBF and Combi | Current feeding type: exclusive breastfeeding from birth (EBF) vs. combination feeding (Combi, any type of supplement with formula) | 15% of the mothers felt guilt, 38% felt stigma, 55% felt the need to defend their choices. Combi mothers had approx. 6 times higher risk of experiencing guilt and dissatisfaction with the feeding method than EBF mothers. EBF mothers more often reported external guilt (from family), whereas Combi mothers reported internal guilt. Intention was not strongly associated with negative experiences after adjustment. Most mothers reported overall satisfaction (94%). Internet was the most common source of information (42%). |
| Alyousefi et al. (2021) | Exclusive breastfeeding, mixed feeding, exclusive formula feeding | WHO definition of EBF (breast milk only, no other foods or liquids except medicine/vitamins). Mixed = breast milk + formula + other foods. Formula feeding = only formula, no breast milk. | Actual feeding practice for the first 6 months: exclusive breastfeeding (EBF), mixed feeding, or exclusive formula feeding | EBF prevalence at 6 months: 28%. Mixed feeding was most common (53.7%). Predictors of successful EBF: antenatal intention, previous breastfeeding experience, not stopping breastfeeding when sick, comfortable breastfeeding around relatives/friends, and social acceptance. Barriers: perceived insufficient milk, lack of suitable public breastfeeding spaces. |
| Fisher et al. (2024) | Breastfeeding, mixed feeding, formula feeding | Self-reported feeding category; mixed = both breast milk and formula; qualitative context | Self-reported actual feeding method in hospital, after discharge, and at interview | Most participants preferred breastfeeding but shifted to mixed feeding due to hospital experiences, low milk supply, bilirubin levels, or pain. Hispanic culture supported breastfeeding but lacked awareness of support resources. |
| Eilers et al. (2020) | Breastfeeding ≥6 months, ≥12 months; supplementation with formula and solids | Based on duration of any breastfeeding and introduction of formula/solids | Breastfeeding initiation, duration ≥6 months, duration ≥12 months | Gap between intention and actual duration; US-born women had the largest drop-off by 2 months |
| Aranda et al. (2025) | Exclusive breastfeeding  Any breastfeeding | Exclusive breastfeeding defined according to maternal report: solids, water, or formula not introduced | Exclusive breastfeeding at 6 months  Any breastfeeding at 12 months | Housing insecurity negatively associated with breastfeeding intentions and practices  No significant association between food insecurity and breastfeeding outcomes Long-term housing insecurity associated with lower breastfeeding at 12 months |
| Symon et al. (2013) | Exclusive breastfeeding, mixed breast/formula feeding, exclusive formula feeding | “Breastfed” included both exclusive breastfeeding and mixed breast/formula feeding (common practice in Scotland) | Actual practice: formula feeding from birth, breastfeeding <3 weeks, breastfeeding 3–8 weeks, breastfeeding >8 weeks (including exclusive or mixed) | Antenatal intention predicted postnatal practice. Highest satisfaction: mothers with no breastfeeding intention who formula fed from birth, and those with high intention who breastfed >8 weeks. Lowest satisfaction: mothers with high intention who breastfed <3 weeks. Common reasons for stopping breastfeeding: pain, latching difficulties, perception of insufficient milk, professional/family pressure, sibling jealousy. Perseverance was key for those who breastfed for longer. |
| Scott et al. (2019) | Exclusive breastfeeding, combination feeding, formula feeding | Defined based on chart documentation of actual feeding method at discharge | Feeding method at discharge (exclusive breastfeeding, mixed, or formula) | 75% of mothers had a negative shift in feeding behavior. The educational intervention did not significantly change breastfeeding rates compared to control. |
| Harrison et al. (2016) | Exclusive breastfeeding, formula feeding, and complementary feeding | Not explicitly described beyond "exclusive breastfeeding," which was defined as only breast milk for up to 6 weeks | Exclusive breastfeeding rate (EBR) at 6 weeks postpartum (reported as 32%) | Only factor significantly associated with EBR was the maternal belief that breast milk alone ensures infant satiety (prevalence ratio: 2.22; 95% CI: 1.34–3.68); sociodemographic variables were not significantly associated with EBR |
| Al-Sagarat AY et al. (2017) | Formula feeding, breastfeeding, not clearly categorized | Self-reported planned and preferred method; items included plans to try breastfeeding or not | none | Majority planned to use formula. Breastfeeding intentions were low. High levels of concern about pain, appearance, and low support. No significant effect of demographic characteristics on intention |
| Davie et al. (2019) | Exclusive breastfeeding, mixed feeding, exclusive formula feeding (measured proportionally) | Proportionate feeding scale (0–100% breastmilk vs. formula in first and last 48 hours postpartum) | Infant feeding practices in first 48 hours and last 48 hours at approx. 2 weeks postpartum, measured as the proportion of breastmilk vs. formula feeds | Antenatal attitudes (IIFAS) and intentions (IFIS) toward breastfeeding were positive and did not differ according to BMI group. Intentions predicted early breastfeeding practice, but BMI did not. Healthy, overweight, and obese women showed comparable early breastfeeding rates (approx. 82–86%). Findings suggest postnatal, not antenatal, factors explain lower breastfeeding rates among women with higher BMI. |
| EA et al. (2014) | Exclusive breastfeeding, predominant breastfeeding, mixed feeding | WHO/UNICEF definitions; EBF = breastmilk only, no other food or liquids in past 24h | Exclusive and full breastfeeding at 3 months postpartum (24h recall) | EBF intention predicted EBF (OR = 1.48) and full BF (OR = 1.34) at 3 months. Knowledge, attitude, and self-efficacy did not predict actual feeding behavior. |
| Hamner et al. (2019) | Breastfeeding only, formula only, both, unsure | Based on self-reported intention and behavior; ≥8 weeks set as duration threshold | Whether intended breastfeeding was initiated and continued ≥8 weeks | Most women who intended to breastfeed did so; however, intention–behavior gaps were larger among Black and Hispanic women. Socioeconomic factors partly explained disparities |
| Johnson et al. (2019) | Exclusive breastfeeding, mixed feeding, exclusive formula feeding | Exclusive breastfeeding: only breast milk, including expressed milk, no formula. Mixed feeding: breast milk and formula.  Exclusive formula feeding: only formula milk. | Postpartum in-hospital feeding (exclusive breastfeeding, mixed, exclusive formula); PPV feeding (same categories) | Prenatal intention predicted postpartum feeding. Women who used contraception types other than LARC or tubal ligation had reduced odds of practicing any breastfeeding (OR = 0.35, p = 0.02). |
| Wang et al. (2010) | Exclusive breastfeeding, partial breastfeeding, formula only | WHO definitions; exclusive = only breastmilk, partial = breastmilk + other food/formula | Initiation, any breastfeeding, and exclusive breastfeeding at various timepoints up to 6 months | Women with strong prenatal breastfeeding intention were more likely to initiate and continue breastfeeding. Strength of intention independently predicted duration of any and exclusive breastfeeding |
| Talbert et al. (2020) | Exclusive breastfeeding, predominant breastfeeding, partial breastfeeding, complementary feeding | WHO definitions: EBF = breastmilk only  Predominant = water/herbal tea  Partial = other milk  Complementary = semi-solids | Exclusive breastfeeding from birth to 6 months. Patterns and deviations documented at nine timepoints | All mothers intended to breastfeed but only 2 practiced EBF for 6 months. Barriers included family advice, traditional medicine, colic perception, and early introduction of porridge. Persistent issues with breastfeeding technique observed. |
| Zhang et al. (2014) | Exclusive breastfeeding, any breastfeeding, formula feeding | Based on duration and exclusivity. Exclusive breastfeeding defined as no other foods/liquids | Actual breastfeeding initiation (yes/no)  Duration of exclusive and any breastfeeding (in weeks) | Formula info from print/web negatively associated with breastfeeding intentions and initiation  Breastfeeding info from magazines positively associated with breastfeeding intentions and initiation  Media exposure influences both intention and behavior |
| McQueen et al. (2015) | Exclusive breastfeeding, combination feeding, formula feeding | Based on Labbok and Krasovec (1990): exclusive, almost exclusive, partial, token breastfeeding | Exclusive and any breastfeeding at 4 and 8 weeks postpartum | Higher BSES-SF scores significantly predicted any and exclusive breastfeeding at 8 weeks postpartum |
| Gage et al. (2012) | Exclusive breastfeeding, formula, mixed feeding | Exclusive breastfeeding = only breastmilk; complementary foods = solids or other liquids | Duration of exclusive breastfeeding; age at introduction of complementary foods | Mothers who relied on written materials practiced EBF for longer and weaned later; those who relied on family/friends practiced EBF for a shorter period. High rate of switching sources of primary info was observed postpartum, especially among younger, less educated mothers. |
| Bahorski et al. (2023) | Exclusive breastfeeding, mixed feeding, formula feeding, complementary foods | Self-reported; EBF = breastmilk only; Mixed = breastmilk + formula/CF; CF = solids before 4–6 months | Exclusive breastfeeding, mixed feeding, CF introduction at 4 months | 45.5% of the mothers deviated from prenatal plans. Higher BMI was associated with deviation (p = .05). Deviation from prenatal plans showed no significant links to race, employment, marital status, or infant-related factors |
| Bramson et al. (2010) | Exclusive breastfeeding  Mixed feeding (breast and formula) Formula only | Defined based on feeding method used during hospital stay, recorded by hospital staff. Categorized as “exclusive breastfeeding” vs. “all others” for analysis. | Exclusive breastfeeding during the maternity hospital stay (as recorded by postpartum hospital staff) | Duration of early skin-to-skin contact showed a dose-response relationship with exclusive breastfeeding: compared to no contact, the OR for ≥1 hour of skin-to-skin contact was 3.145 (95% CI: 2.905–3.405) for exclusive breastfeeding. |
| Street et al. (2011) | Breastfeeding, formula feeding | Defined based on maternal report; initiation = breastfeeding started within first days postpartum | Breastfeeding initiation at birth | More positive feeding attitudes predicted breastfeeding initiation. IIFAS scores were correlated with feeding intention and actual behavior. Formula-feeding intention group showed lower initiation rates. |
| Wallenborn et al. (2019) | Exclusive breastfeeding, mixed feeding, formula feeding (measured using IFPS II categories) | Exclusive breastfeeding defined per AAP: breastmilk only, no other foods or drinks except medicines/vitamins | Breastfeeding duration (weeks) and exclusive breastfeeding duration (weeks) | Workplace support was positively associated with longer breastfeeding duration, but the effect was mediated mainly by self-efficacy. Self-efficacy accounted for 40.8% of the total effect (p=0.032). Intention also predicted duration, but indirect effects of intention were not statistically significant after adjustment. |
| Ware et al. (2021) | Exclusive breastfeeding (at breast), expressed breast milk, mixed feeding (breast + formula), formula only | Any breast milk feeding categories recorded separately (breast only, expressed, mixed with formula, or formula only) | Reported practices at meetings: breast milk at breast only, expressed milk, mixed with formula, or formula only | 67 meetings with 158 total participants have been conducted since 2017. Prenatal intention for breast milk only: 60%. Actual feeding at meetings: breast milk only, 55%;  formula only, 18%. Breastfeeding initiation in Avondale neighborhood increased from 43% (2017) to 55% (2020). Positive qualitative feedback highlighted support, empowerment, and leadership growth among “Champion Moms.” |
| SuÃ¡rez-Cotelo MDC et al. (2019) | Exclusive breastfeeding (EBF), artificial feeding (AF), mixed feeding (breast + other liquids/foods, including formula) | EBF = only breast milk; AF = only artificial milk or with solids; mixed feeding = breast milk plus other foods/liquids including formula | Feeding practices at 6 weeks, 16 weeks, and 6 months postpartum (EBF vs. AF/Mixed F) | 90.4% of women intended to practice EBF, but the actual proportion who did dropped to 63.9% (6 weeks), 52% (16 weeks), and 28.2% (6 months). Knowledge level: regular, 55.5%; good, 25%; poor, 19.5%. Higher knowledge level was correlated with higher level of intention and greater likelihood of maintaining EBF postpartum. Socio-demographic factors (age, parity, education) influenced knowledge. |
| Zhang et al. (2022) | Exclusive breastfeeding, formula feeding, mixed (breast + formula) | Exclusive BF = only breast milk, no formula; Any BF = breast milk with or without formula | 1) Exclusive breastfeeding through 5 months (discrete)  2) Days of exclusive breastfeeding up to 7 months (continuous)  3) Any breastfeeding through 11 months (discrete)  4) Days of any breastfeeding up to 13 months (continuous) | Prenatal perception that WIC recommended breastfeeding only predicted significantly lower risk of stopping exclusive breastfeeding through 5 months (HR=0.83), 7 months (HR=0.78), and any breastfeeding through 11 months (HR=0.80) and 13 months (HR=0.82). Effect persisted even after controlling for IFI in some models. Strong IFI was also a significant independent predictor of longer exclusivity/duration. |
| Radzyminski et al. (2016) | Exclusive breastfeeding, formula feeding, combination feeding | Self-identified; combination feeders often mostly formula-fed (mean, 70%) | Breastfeeding only (38%), formula only (16%), combination feeding (46%) | Mothers’ decisions were infant- or self-centered. Combination feeding aligned with breastfeeding identity. Lack of information and social pressure influenced formula use. |
| Onifade et al. (2023) | Exclusive breastfeeding, expressed breast milk, mixed feeding, formula feeding | Breastfeeding = any form (direct or expressed milk). Exclusive breastfeeding defined based on antenatal records intention category. | Initiation, prevalence of any breastfeeding, breastfeeding duration (median, 35 days), difficulties with breastfeeding | 72.8% of mothers intended to exclusively breastfeed; 83.9% of infants were ever breastfed; median breastfeeding duration was 35 days (IQR, 14–150). A third of the mothers reported breastfeeding problems, especially latching and milk supply. Initiation rates were similar to those observed among non-remote First Nations groups but the duration was substantially shorter. |
| Nazirah et al. (2020) | Exclusive breastfeeding, partial feeding (includes formula, water, or other foods) | EBF based on WHO definition: only breast milk, no water/formula/other foods | Exclusive breastfeeding at 1, 3, and 6 months postpartum | Mean IFI score = 12.8 (SD, 3.7). At 6 months, only 42.1% were exclusively breastfeeding. Significant association between prenatal intention and 6-month EBF practice (OR 2.19, 95% CI 1.01–4.76). No significant association at 1 or 3 months. |
| Thomson et al. (2022) | Exclusive breastfeeding, mixed feeding, formula feeding (reported retrospectively in interviews) | Breastfeeding = any feeding at breast or using expressed milk Discontinuation = stopping all breastfeeding by 8 weeks | Breastfeeding continuation at 8 weeks postpartum (exclusive or mixed vs. discontinued) | Two typologies: “Disappointed” (n=7) – stopped breastfeeding early, often younger, White-British, mixed-feeding intention, negative professional/personal support, infant complications, unexpected hospital top-ups; “By hook or by crook” (n=17) – continued breastfeeding despite problems, more proactive help-seeking, positive support, accessed wider community support. |
| Xu et al. (2022) | Exclusive breastfeeding, any breastfeeding, discontinued | WHO definitions; EBF = only breast milk, no other liquids/solids; any BF includes partial | Exclusive and any breastfeeding at 6 and 12 months | Knowledge and intention predicted longer breastfeeding. Actual EBF rate at 6 months was very low (4%). Staying at home, social support, and exposure to breastfeeding women predicted longer duration. |
| Addicks et al. (2019) | Exclusive breastfeeding, mixed feeding, formula only | Based on maternal report: exclusive = only breastmilk; mixed = breastmilk + formula | Breastfeeding initiation, exclusivity at 1 month postpartum | Intervention group showed greater increases in confidence and importance scores; no significant difference in actual breastfeeding rate at 1 month |
| Tully et al. (2014) | Exclusive breastfeeding, mixed feeding, formula feeding | Self-reported; defined based on mothers’ responses and practices on the ward | 73% of those who intended to breastfeed exclusively breastfed during hospitalization | Mothers who reported self-benefits in motivation were more likely to maintain exclusive breastfeeding. Barriers included pain, fatigue, infant mucus, nighttime difficulty, and lack of support. |
| Francis et al. (2024) | Any breast milk, only breast milk, mixed (breast milk + formula), formula only | Any breast milk = ever breastfed/given breast milk  Only breast milk = no formula before 6 months (NB: full WHO EBF definition was not applied in the survey [i.e., solids/liquids not captured]) | Achievement of intention:  (1) any breast milk (ever breastfed / breast milk provided)  (2) only breast milk (initiated and did not introduce formula until 6 months) | 88% intended to feed any breast milk (99% achieved the feeding goal); 77% intended to feed only breast milk (51% achieved the feeding goal). Household food insecurity not associated with intention but was negatively associated with achievement of exclusive breast milk feeding (adjusted OR, 0.54). Multiparity increased the odds of achieving intention. Planning to pump was negatively associated with achievement. |
| LeMoine et al. (2022) | Breastfeeding, combination feeding, formula only. | Based on maternal report of intent and discharge status; validated that intent at discharge corresponded to actual infant feeding 99.7% of the time. | Feeding plan reassessed at hospital discharge; validated as proxy for actual feeding status. Outcomes: breastfeeding success (intended & was breastfeeding at discharge), unsuccessful (intended but was formula feeding at discharge), never (no intention & no breastfeeding at discharge), unexpected (no intention but breastfed at discharge). | Strongest predictor of breastfeeding at discharge was intent to breastfeed at admission (aOR, 7.16; 95% CI, 6.55–7.84). Success more likely among mothers who were married, privately insured, had higher education, were older. Being African Americans strongly predicted non-intention and conversion to formula feeding (aOR, 0.68). Longer hospital stay was associated with maintaining breastfeeding. |
| Lundberg et al. (2012) | Breastfeeding, water, artificial milk, supplementary foods (sweet/salty powders, rice soup, porridge). | Based on mothers’ narratives (exclusive breastfeeding not practiced; combination feeding and supplementation common). | Described practices: giving water after breastfeeding, discarding colostrum, early use of artificial milk, introduction of supplementary foods around 4 months, mixed feeding. | Mothers had positive attitudes; believed breast milk is best, creates bonding, prevents illness. Exclusive breastfeeding was rare; colostrum was often discarded. Influences included cultural beliefs, traditional diet/rituals, advice from family elders, husband’s support, work obligations, media/advertising of formula, and lactation problems. Mothers saw infant feeding as a learning process.  Duration of intended breastfeeding was usually 12–24 months, but supplementation introduced much earlier |
| Anderson et al. (2024) | Exclusive breastfeeding, any breastfeeding | Mother-reported duration | Duration of exclusive and any breastfeeding | Stronger intention predicted longer breastfeeding, especially among women with low BMI |
| Rafizadeh et al. (2019) | Not directly categorized; breastfeeding behavior was a composite latent variable | WHO-based definitions for exclusive breastfeeding; behavior assessed using six indicators | Breastfeeding behavior as a latent construct (e.g., EBF, initiation time, bottle/pacifier use) | Breastfeeding intention strongly predicted behavior (β = 0.857); attitude, social support, and self-efficacy predicted intention |
| Nelson et al. (2018) | Breastfeeding | Duration in months (self-reported) | Actual breastfeeding duration | One-third shortened their duration intention postpartum; shorter actual duration was observed in this group |
| Mancini et al. (2016) | Exclusive breastfeeding vs. exclusive formula feeding | Breastfeeding: mothers who were exclusively breastfeeding.  Non-breastfeeding: mothers who were exclusively formula feeding | Exclusive breastfeeding vs. exclusive formula feeding at 8–26 weeks | No significant difference in body image (MBSRQ-AS) or eating attitudes (EAT-26) between the breastfeeding and non-breastfeeding mothers  Mothers with positive intention to breastfeed had lower (better) EAT-26 scores than those with no intention, but were not significantly different from the “undecided” mothers  Positive intention to breastfeed was a strong predictor of actual breastfeeding (97% of breastfeeding mothers had expressed positive intentions). |
| Springall et al. (2023) | Survey response options:  fully breastfeeding; breastfeeding + expressed breast milk (EBM); EBM; breastfeeding + formula; breastfeeding + EBM + formula; EBM + formula; fully formula feeding. For analysis, categories were combined into “any” breast milk and “only” breast milk at 3 months, plus initiation. | “Any” breast milk: infant received breast milk regardless of the introduction of other milk substitutes, liquids, or foods. “Only” breast milk: breast milk was the only form of milk (other liquids/foods may have been introduced); not equivalent to exclusive breastfeeding. | Primary outcomes: breastfeeding initiation; breastfeeding maintenance at 3 months.  Initiation: 96% (95% CI, 0.93–0.98) “Any” breast milk at 3 months: 71% (95% CI, 0.65–0.78) “Only” breast milk at 3 months: 48% (95% CI, 0.41–0.55) | 87% of the participants received the culturally specific caseload midwifery model.  Initiation: 96% at 3 months; 71% for “any” breast milk and 48% for “only” breast milk.  Intention to breastfeed for ≥6 months increased the odds of breastfeeding at 3 months (“any” breast milk: adj OR = 2.69, 95% CI = 1.29–5.60; “only” breast milk: adj OR = 2.22, 95% CI = 1.20–4.12).  Not smoking during pregnancy increased the odds of breastfeeding (“any”: adj OR = 2.48, 1.05–5.86; “only”: adj OR = 4.05, 1.54–10.69).  Lower education (≤diploma) reduced the odds of breastfeeding (“any”: adj OR = 0.36, 0.13–0.98; “only”: adj OR = 0.50, 0.26–0.96).  Government benefits as main household income reduced odds the of “any” breastfeeding (adj OR = 0.26, 0.11–0.58).  Most important reason for stopping breastfeeding was perceived insufficient milk / uncertainty baby had enough (32% of those who stopped). |
| Alkhaldi et al. (2023) | Captured intention (“breastfeed,” “exclusively breastfeed” willingness); early initiation timing; no detailed multi‑category feeding taxonomy used | IIFAS (17–85): positive to BF (70–85), neutral (49–69), positive to formula (17–48). “Exclusive breastfeeding” defined as only breast milk | Not a primary outcome. Descriptive peripartum metrics reported: initiated BF on day 1 (43.2%); did not initiate before discharge (28.6%); received any BF counselling (40%) | Mean IIFAS score 65.0±7.2 (neutral, upper bound) Positive BF attitude: 24.3%.  Intention to BF: 95.7%  Willing to EBF: 35.2%.  Determinants of positive attitude (logistic regression): highest income >651 JD (OR, 14.77; 95%CI, 2.25–99.64); income 301–450 JD (OR, 4.88; 1.12–23.8); willingness to EBF (OR, 3.41; 1.35–8.63) |
| Mirkovic et al. (2014) | Breastfeeding (exclusive/predominant not distinguished) | Self-reported breastfeeding cessation age | Whether actual duration was ≥3 months | Returning to full-time work before 3 months postpartum was associated with higher odds of not meeting breastfeeding intentions |
| Nommsen-Rivers et al. (2010) | Number of “breastfed well” episodes in first 24–48 h; volumes of formula supplemented (0–48 h); no full taxonomy beyond these | Delayed OL >72 h; IBFAT (0–12; <11=suboptimal); Faces Pain Scale–Revised for nipple/breast pain; BMI: <25 (normal), 25–29.9 (overweight), ≥30 (obese) | Primary outcome: Delayed onset of lactogenesis (OL) = maternal perception of “noticeably fuller” breasts >72 h postpartum  Key numbers: median OL, 68.9 h; 44% reported delayed OL | Independent risk factors for delayed OL (adjusted): • Maternal age ≥30 y • Overweight/obese BMI • Infant birth weight >3600 g • No nipple discomfort (days 0–3) • Infant failed to “breastfeed well” ≥2 times in first 24 h  Postpartum edema was significant factor in an alternate model (when BMI excluded). Cesarean and labor-related factors attenuated after adjustment. |
| Raissian et al. (2018) | Exclusive BF, any BF, no BF | “Exclusive BF” = only breastmilk at the timepoint | Exclusive BF at 2 months (primary); any BF at 2 months; also included 6‑month variants | After adding prenatal intention, BF–health associations largely attenuated; intention remained protective. “Intend+no BF” ≈ “Intend+BF” on outcomes. Intending mothers had more nutrition knowledge and more info sources. |
| Donnan et al. (2013) | Only breast milk, both breast and formula milk, only formula milk | Self-reported feeding type every 2 weeks via SMS | Initiation and cessation of any and exclusive breastfeeding up to 16 weeks | High intention (TPB) and attitude (IIFAS) predicted initiation and longer duration. Prior breastfeeding and professional occupation were also associated with breastfeeding. |
| Miho Ito et al. (2022) | Breastfeeding only, mixed feeding | Feeding method at hospital discharge | Breastfeeding method at discharge (exclusive or mixed) | Eight categories were identified: hopeful intention, shallow knowledge, unfounded confidence, anxiety, and postnatal gap in expectations |
| Summary | EBF defined (WHO-based): 27%; Mixed feeding defined: 22%; Formula-only defined: 15% | — | Median follow-up: 6 months | — |

EBF, exclusive breastfeeding; WHO-based, aligned with WHO definition; self-defined, author-defined criteria.
