# Supplementary material for: Defining and measuring maternal intentions and practices regarding infant feeding: a scoping review
Source: BMC Pregnancy Childbirth. 2026 Feb 11;26:286. doi: 10.1186/s12884-026-08768-0 (PMC12998363; doi:10.1186/s12884-026-08768-0)
Supplement: Supplementary file 2 — Supplementary Material 2. [file 12884_2026_8768_MOESM2_ESM.docx]

Appendix 1. Full Search Strategy

**PubMed**

(“breastfeeding” OR “breast feeding” OR “exclusive breastfeeding” OR “partial breastfeeding” OR “formula feeding” OR “bottle feeding” OR “mixed feeding”) AND (“feeding intention” OR “intended feeding” OR “feeding decision” OR “feeding practice” OR “actual feeding” OR “infant feeding” OR “infant nutrition”)

**CINAHL**

(("breastfeeding" OR "breast feeding" OR "exclusive breastfeeding" OR "partial breastfeeding" OR "formula feeding" OR "bottle feeding" OR "mixed feeding")

AND("feeding intention" OR "intended feeding" OR "feeding decision" OR "feeding practice" OR "actual feeding" OR "infant feeding" OR "infant nutrition"))

**Ichushi-Web (Japan Medical Abstracts Society)**

（母乳育児 OR 混合栄養 OR 人工栄養 OR 授乳方法） AND （意図 OR 意思決定 OR 実践 OR 希望 OR 母親）
