# Supplementary material for: Defining and measuring maternal intentions and practices regarding infant feeding: a scoping review
Source: BMC Pregnancy Childbirth. 2026 Feb 11;26:286. doi: 10.1186/s12884-026-08768-0 (PMC12998363; doi:10.1186/s12884-026-08768-0)
Supplement: Supplementary file 3 — Supplementary Material 3. [file 12884_2026_8768_MOESM3_ESM.docx]

Appendix 2. Data Extraction Form

Section A. Study Identification and Characteristics

| Item | Description |
| --- | --- |
| Study ID | First author, year |
| Country / Region | Country or geographic region |
| Study design | Quantitative / Qualitative / Mixed-methods |
| Study setting | Hospital, community, national survey, etc. |
| Sample size | Number of participants |
| Population characteristics | General maternal population / specific population (if applicable) |
| Infant age at assessment | e.g., birth, 1 month, 6 months |

Section B. Conceptualization and Measurement of Maternal Feeding Intention

| Item | Description |
| --- | --- |
| Presence of feeding intention assessed | Yes / No |
| Conceptual definition of intention | Author-defined description |
| Type of intention measure | Single-item / Multi-item scale |
| Name of instrument (if applicable) | e.g., Infant Feeding Intention Scale |
| Theoretical framework | TPB, self-efficacy, none specified |
| Timing of intention assessment | Antenatal / Postnatal (specify time point) |
| Response format | Categorical / Likert scale / VAS / Open-ended |

Section C. Conceptualization and Measurement of Infant Feeding Practices

| Item | Description |
| --- | --- |
| Feeding categories assessed | Breastfeeding / Mixed feeding / Formula feeding |
| Definition of exclusive breastfeeding | WHO-based / Author-defined |
| Definition of mixed feeding | Author-defined description |
| Definition of formula feeding | Author-defined description |
| Measurement indicator | Initiation / Duration / Proportion / Mode |
| Data source | Self-report / Medical record / Registry |
| Timing of practice assessment | e.g., discharge, 1 month, 6 months |

WHO, World Health Organization
